# Vaccination, politics and COVID-19 impacts

**DOI:** 10.1186/s12889-021-12432-x

**Published:** 2022-01-14

**Authors:** Don Albrecht

**Affiliations:** Utah State University, Logan, Utah USA

## Abstract

The development of safe and effective COVID-19 vaccines provides a clear path to bring the pandemic to an end. Vaccination rates, however, have been insufficient to prevent disease spread. A critical factor in so many people choosing not to be vaccinated is their political views. In this study, a path model is developed and tested to explore the impacts of political views on vaccination rates and COVID-19 cases and deaths per 100,000 residents in U.S. counties. The data strongly supported the model. In counties with a high percentage of Republican voters, vaccination rates were significantly lower and COVID-19 cases and deaths per 100,000 residents were much higher. Moving forward, it is critical to find ways to overcome political division and rebuild trust in science and health professionals.

## Introduction

After initially appearing in late 2019, the COVID-19 virus spread around the world, causing devastation everywhere. By September, 2021, the known worldwide death toll exceeded 4.6 million. In the U. S, more than 40 million people had tested positive for the virus and the death toll was approaching 700,000. In addition to the physical health consequences, the economic, social, mental health, education and other impacts from the pandemic have been substantial [1–4].

The emergence of COVID-19 was not especially surprising. Scientists and health experts have long warned of potential devastating impacts of a pandemic resulting from the emergence of a new disease for which humans have little or no resistance [5–10]. From a historical perspective, COVID-19 is but the latest in a series of infectious diseases that have devastated human communities. For example, in the fourteenth Century, the Bubonic Plague killed perhaps 20 million people which was about one-third of the population of Europe at the time [11]. The Spanish Flu pandemic of 1918 killed an estimated 50 million people [12]. When Europeans began travelling to the western hemisphere, they brought diseases that had devastating impacts on native populations [13]. It is estimated that 55 million Native Americans died from diseases introduced from Europe within the first century of contact. A majority of the population in some locations were killed [14].

With the COVID-19 pandemic, however, there was hope that through the development of vaccines, the disease could be controlled and the world returned to normal much quicker and with less damage than had occurred with previous pandemics [15]. Prior to COVID-19, the time needed to develop a safe and effective vaccine had been measured in decades [16]. However, resulting from years of basic scientific research that led to a greater understanding of human cells, how viruses attack these cells, and how defenses to the virus can be implemented [17], safe and effective vaccines were developed in record time. The genetic sequence of the virus causing COVID-19 was published on January 11, 2020, and by March 16, 2020 human clinical testing of a vaccine began [18]. Nine months later, in December 2020, the first vaccines were being delivered. Results clearly show COVID-19 vaccines to be very safe and effective [19, 20]. If widely used, these vaccines could bring the disease under control [21]. For example, in Portugal and other countries where nearly all eligible residents are vaccinated, COVID-19 cases have become rare.

Months after the COVID-19 vaccination process began, however, the pandemic continued to rage in many countries around the world. This is largely because the proportion of the population vaccinated against the disease in these countries is insufficient to reach ‘herd immunity’. In the United States, for example, nearly 175 million persons were fully vaccinated against COVID-19 as of September 1, 2021. This was only 61.6% of eligible persons (those persons age 12 and over at the time), and only 52.7% of the total population. Two major factors explain inadequate vaccination uptake. First, primarily in developing counties, the number of shots available has been insufficient and the capacity to reach some segments of the population has been lacking [22]. In some developed countries, however, the major impediment has been large numbers of people, especially persons with certain characteristics, choosing not to be vaccinated [23–27]. While there has always been vaccine hesitancy [28–30], COVID-19 vaccine resistance appears different because of deep political underpinnings. Understanding why people are refusing to be vaccinated and the role of political views in these decisions is a question of utmost significance since these choices have severely hampered efforts to control the COVID-19 virus.

The goal of this manuscript is to improve our understanding of factors related to COVID-19 vaccination decisions and the consequences of these decisions. While a number of studies have explored factors related to vaccination hesitancy [24–26, 30–32], this study focuses on the role of political views in explaining variations in actual vaccination rates across U.S. counties. In addition, analysis is conducted on the relationship between political views, vaccination rates and per capita COVID-19 cases and deaths in U.S. counties. This manuscript continues with a discussion of vaccination resistance. Following this, a research model is developed and then data analyzed to test the model.

### Vaccination resistance

For generations, Smallpox was among the deadliest of diseases of humans. Over the centuries, millions of people were killed by this terrible disease. Smallpox was the primary killer of native populations in the Americas after European contact. Periodic devastating Smallpox outbreaks occurred in cities throughout the world on a somewhat regular basis. Then in the late 1700s, Edward Jenner developed a process to combat Smallpox by intentionally introducing cowpox into humans. By contracting cowpox, a much less severe disease, people developed an immunity to the deadly Smallpox. This process alone saved millions of lives. With later improvement to Smallpox vaccines, the World Health Organization declared in 1980 that Smallpox, one of the greatest killers of all time, had been eradicated from the earth [11].

The development of other vaccines has helped dramatically extend human life expectancy and well-being. In the 1950s Jonas Salk developed a vaccine for Polio. Until that time, Polio was killing large numbers of people and crippling even more. Since the development of the Polio vaccine, the impacts of the disease have been dramatically reduced. Vaccines were developed for other significant diseases including measles, mumps, rubella, tetanus, typhoid, diphtheria and pertussis. Vaccines have saved more lives than any other medical technology [33].

Despite the obvious fact that vaccines save lives and reduce human suffering [34], there has been opposition to vaccination since the time of Jenner [35]. This opposition has become more organized and vibrant in recent years, with help from the Internet and social media [30, 36]. A critical event was an article published in 1998 that purported a link between the MMR (measles, mumps, and rubella) vaccine and autism. Later it was found that the research was faulty and the article was retracted in 2010. The damage, however, had been done and a strong “anti-vax” movement was growing throughout the world. The movement was greatly enhanced by tweets from Donald Trump both before his election and after he became president [31]. The consequences are profound, and vaccination rates have been declining around the world [37].

A significant consequences of declining vaccination rates is that some diseases that were under control are reemerging [38, 39]. Measles provides a case-in-point. In the decade prior to 1963, 3–4 million people in the U.S. (mostly children) contracted measles each year. Nearly all children had been infected by the disease before age 15. About 400–500 people died from measles each year. For thousands more, measles led to a more dangerous illness, such as encephalitis. After a vaccine for measles was released in 1963, children were vaccinated throughout the world, and the number of cases plunged. By 2000, measles had been eliminated from the U.S. Then, however, the disease returned. Unvaccinated international travelers in countries where the disease was not yet eliminated contracted the disease and brought it back into the U.S. The disease has been able to spread because there are now so many people who have not been vaccinated. In 2019 there were about 1300 measles cases in the U.S. [39]

Opposition to the COVID-19 vaccine emerged immediately after it was announced that vaccine developments were under way. As preparations were being made for the release of COVID-19 vaccinations, social media posts presented a range of falsehoods about the vaccines, including claims that COVID vaccines would alter DNA, negatively affect fertility, or that the government was injecting microchips into people so that their behavior could be monitored [40]. Many people maintained that whether or not they were vaccinated was a personal choice that should not be mandated by the government. For these and other reasons, large numbers of people are resistant to receiving COVID-19 vaccination. People with certain characteristics are much more susceptible to such arguments and thus more likely to choose not to be vaccinated than others. Of special interest for this study is the role of political views in influencing vaccination rates and consequently COVID-19 impacts.

### Research model

**Fig. 1:**
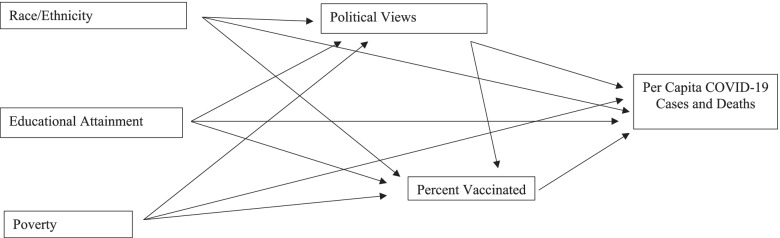
Research Model Used in the Analysis

The proposed research model utilized in this study is presented in Fig. 1. This model suggests that three exogenous factors (race/ethnicity, educational attainment, and poverty) help explain variations in political views. Political views then are expected to strongly influence vaccination rates, which, in turn, influence the severity of COVID-19 in U.S. counties as measured by COVID-19 cases and deaths per 100,000 residents. The exogenous variables and political views are also expected to have both direct and indirect impacts on COVID-19 cases and deaths per 100,000 residents. Each portion of the model is described below.

### Exogenous variables

Three exogenous variables were selected for use in this analysis. These variables were chosen because previous research has shown them to be strongly related to political views [41–44]. These variables are also expected to have direct effects on both vaccination rates and COVID-19 cases and deaths. The exogenous variables are then expected to indirectly influence vaccination rates and COVID-19 impacts through their relationship with political views.

#### Race/ethnicity

Analysis of the 2016 and 2020 presidential elections found that a large proportion of the non-college educated non-Hispanic white population voted for Donald Trump [41, 45–47]. Trump’s domination of the white vote is part of a trend where the political parties have become increasingly different on racial issues. Since passage of the Civil Rights Act in 1964, Democrats have emphasized policies supported by minorities such as affirmative action, fair housing, school integration, higher minimum wages, and the elimination of discrimination in the workplace. As a consequence, minorities have voted heavily Democrat ever since [48, 49].

With the pro-minority platform of Democrats, Republicans recognized an opportunity to pull away some white voters who had previously voted Democrat [50]. Beginning with the Nixon campaign of 1968, Republicans implemented a “Southern strategy” that made an appeal to racial conservatism [51–55]. Racial conservatism maintains that minorities no longer face discrimination and minority disadvantages are due to their poor work ethic and failure to embrace American values. At the same time, poor whites are told by Republicans that their circumstances are made worse because so many resources are diverted to programs that benefit undeserving minorities [56–58].

To a large extent, Republican plans have worked. The Republican party now has strong support from the white working class [44]. Subsequently, race/ethnicity has become an important predictor of voting behavior [59]. The relevance of the race/ethnicity variable was especially high in both the 2016 and 2020 elections as Trump ran campaigns that effectively motivated white voters around their racial identity [45]. Trump’s campaign had clear racial undertones and studies have shown that this helped him receive a higher proportion of the white vote than Republican candidates in previous elections [60]. A study of Iowa voters [46], found that race, not economics was the critical factor motivating Trump voters. Smith and Hanley [47] concluded Trump’s supporters voted for him because they shared his racial prejudices, not because they were financially stressed.

For this study, it is thus expected that there will be a positive relationship between the percent of residents in a county that are non-Hispanic white and the percent voting for Trump in the 2020 presidential election. Further, it is expected that counties with high proportions of non-Hispanic white residents will have lower vaccination rates, largely because of the indirect effects of political views. Additionally, low vaccination rates are expected to result in more severe COVID-19 impacts in counties with large non-Hispanic white populations, a finding supported by existing studies [61].

#### Educational attainment

In recent elections, persons with higher levels of educational attainment have tended to vote Democrat. Several factors may explain this tendency including views on environmental issues, cultural views and attitudes about science. First, highly educated persons are more likely to support the environmental platform of Democrats [62–64]. In contrast, Republicans maintain that environmental protection policies tend to harm the economy. Second, persons with higher levels of educational attainment are more likely to be supportive of the inclusive multicultural society emphasized by Democrats [65]. Republican support generally comes from persons with less education who are threatened by these cultural ideals and the changes they represent [57]. Finally, persons with higher levels of educational attainment are more likely to be troubled by science denial. Science denial is more prevalent among Republicans than Democrats [66–70]. Science denial is especially relevant in this study about vaccinations, which have been developed and are strongly supported by the scientific community. Thus, in counties with high levels of educational attainment, it is expected that the proportion of votes for Trump will be reduced. Further, with greater trust in science, educational attainment levels are expected to be positively related to vaccination rates, an expectation supported in a study by Sun and Monnat [27]. Combined, these factors are expected to result in lower per capita cases and deaths from COVID-19 in counties with high levels of educational attainment.

#### Poverty

Poverty rates are much higher in communities with large minority populations [71]. Consequently, since minorities are more likely to vote Democrat, it is expected that there will be an inverse relationship between poverty rates in a county and the percent voting for Trump in that county. It is further expected that there will be an inverse relationship between poverty rates and vaccination rates, in spite of political views [72]. Reasons for low vaccination rates in high poverty communities include language barriers and a lack of trust in health experts [73, 74]. Thus, Moore et al. [75] found significant levels of vaccine resistance in low-income black communities in the South. Finally, research has found that high poverty communities have significantly higher rates of COVID-19 cases and deaths than communities with lower poverty rates [61, 76–78]. Often persons in poverty are living in crowded and unsanitary conditions that enhance disease spread. Additionally, persons living in poverty are more likely to have underlying health conditions and often have inadequate health care [79, 80]. The relationship between poverty levels and both vaccination rates and per capita COVID-19 cases and deaths is expected to be indirectly impacted by political views.

### Political views

The widespread use of safe and effective COVID-19 vaccines represents a clear path to end the COVID-19 pandemic. Unfortunately, vaccination levels have not been high enough to stop disease spread. A critical factor in vaccine resistance is political views [27]. The role of politics has had a critical impact on COVID-19 responses to the pandemic in the U.S. from the outset. From the beginning, Democrats were much more likely than Republicans to take the threat of the virus seriously and to support efforts to control it [81, 82]. Thus, early research found that counties with a higher share of Republican voters tended to have lower perceptions of the dangers of COVID-19, and these perceptions led to riskier behavior [83, 84]. States with more Republican voters were more resistant to stay-at-home orders [85]. In more religious states, which tend to be heavily Republican, people were found to be more mobile during the pandemic despite recommendations to stay home [86]. Perry et al. [87] found that Christian nationalism, which has strong ties to the Republican Party, was related to many of the far-right responses to COVID-19, including unfounded conspiracy theories. As a consequence of these views, Albrecht [61] found that counties with a high proportion of Trump voters had more per capita cases and deaths from COVID-19 than counties with fewer Trump voters.

Politics have also greatly influenced views about COVID-19 vaccines. Research has confirmed a strong relationship between political views and vaccination uptake in both the United States and other countries. These studies have consistently found political conservatives to be more vaccine resistant [23, 26, 27, 88, 89]. Events like those occurring in Moroni in rural Utah on April 30, 2021 are characteristic of opposition to vaccines in conservative communities. In what was advertised as a “Night of Liberty,” the world’s largest syringe (made of wood) was burned as hundreds of people watched and cheered to protest “Medical Tyranny” [90]. A big issue in vaccine hesitancy among conservatives is science skepticism [91].

Political differences relative to views about COVID-19 and vaccinations to combat the disease started at the top. From the beginning, the severity of the pandemic was downplayed by President Trump. Trump talked about how the virus would magically disappear. He then claimed that the virus would be eliminated by warmer spring weather. For months, he argued that we were turning the corner and that the disease wasn’t that bad anyway. He recommended ways of addressing the disease that lacked scientific merit. Trump held political rallies where thousands of people gathered, most not wearing masks. Reacting to shut down policies intended to slow disease spread, Trump tweeted messages such as “Liberate Michigan” [92].

Conservative opposition to vaccines was enhanced by the support of Donald Trump. Over the years, Trump has sent many tweets with anti-vax and pro-conspiracy theory themes. For example, in 2014 he tweeted, “Healthy young child goes to doctor, gets pumped with massive shot of many vaccines, doesn’t feel good and changes – AUTISM. Many such cases.” On September 2, 2015 he tweeted, “I am being proven right about massive vaccinations – the doctors lied. Save our children and their futures!” A study by Hornsey et al. [31] found that these statements had an effect and that Trump voters were more likely to express vaccine hesitancy, distrust medical authorities, and believe conspiracy theories about COVID-19 vaccines.

Beyond the president, other political leaders and media outlets sent divergent messages on COVID-19. Again, Republicans and the right-wing media tended to downplay the threat of the disease and express opposition to steps intended to prevent spread [93]. Fridman et al. [94] found a critical factor in vaccine resistance was exposure to right-wing media. With support from Republican leaders and the right-wing media, protests were held throughout the country in opposition to mask mandates, business and school closures, and vaccination mandates. In many communities, wearing a mask or getting a vaccine became a political statement, with many Republicans arguing that these actions violated their individual freedoms and were unnecessary anyway. The consequence was increased levels of virus spread in Republican-dominated counties. A study from early in the pandemic found that counties where Trump received a higher proportion of the vote were initially safer from the virus, but this changed as the pandemic progressed, and these counties then experienced severe impacts [95]. Research shows that a likely reason for the initial safety of Trump-leaning counties from the disease is that they tend to be more rural where people are naturally social distanced and less likely to be reliant on mass transit, conditions which enhance virus spread [61]. This same study found a positive relationship between the percent voting for Trump in a county and the severity of the pandemic in that county. In this study, we expect an inverse relationship between the percent voting for Trump and vaccination rates. Lower vaccination rates are expected to lead to higher rates of COVID-19 cases and deaths.

### COVID-19 cases and deaths

The ultimate dependent variables for this study are COVID-19 cases and deaths per 100,000 residents. Disease rates are expected to be lower in counties where vaccination rates are higher, where the percent voting for Trump is lower, where the percent non-Hispanic white population is lower, where educational attainment levels are higher and where poverty rates are higher.

## Methods

The county is the unit of analysis for this study. Counties are relatively small geographic units for which data are available for all of the variables utilized. The analysis is based on 3112 counties for which data are available on all of the variables used in the analysis [61]. The dependent variables are the number of COVID-19 cases and deaths per 100,000 residents by county between March 1, 2021 and September 1, 2021. This time period was chosen because it is a six-month period between the time when vaccines became readily available and a time-period long enough so that the consequences of vaccine use or the lack thereof to be apparent. To measure the dependent variables, county level data were obtained from the New York Times dataset [96]. This dataset provides the cumulative number of COVID-19 cases and deaths for each county in the U.S. on a daily basis. New York Times data is obtained from state, regional and county sources on a continual basis. New York Times data is virtually identical to data from other sources since all data providers get their information from the same places. The advantage of the New York Times dataset is that it is available to the general public and can be easily downloaded.

For this study, the total number of COVID-19 cases and deaths in each county were downloaded on three different dates - May 1, 2020, March 1, 2021 and September 1, 2021. Data from all three dates are used in categorical tables showing the progression of the disease over time in counties that vary by political views and vaccination rates. The dependent variables used in the path analysis were then created by subtracting COVID-19 cases and deaths in each county on March 1, 2021 from COVID-19 cases and deaths on September 1, 2021. This number is then divided by the total population of that county as reported by the 2014–2018 American Community Survey and multiplied by 100,000.

Vaccination rates are based on CDC (Center for Disease Control and Prevention) data as downloaded on September 1, 2021. This measure shows the percent of persons in each county who were fully vaccinated. At this time, children 11 and younger were not eligible to be vaccinated. Political views are measured by the percent of votes for Donald Trump in each county in the 2020 presidential election. County level voting data were downloaded from the New York Times [97] and determination was made of the percent of voters in each county that cast their ballot for Donald Trump in the 2020 presidential election. Data for the three exogenous variables were obtained from the 2014–2018 American Community Survey. Race/ethnicity is measured by the percent of residents in each county that are non-Hispanic white; educational attainment is determined by the percent of persons aged 25 and older in each county with a college degree; and poverty is measured by the percent of person in each county living below the census defined poverty line.

All methods were performed in accordance with relevant guidelines and regulations. The analysis begins with a categorical overview of the relationship between both political views and vaccination rates on COVID-19 cases and deaths over time. These tables also show the exogenous variables by political views and vaccination rates. This is followed by an analysis of the path model presented in Fig. 1.

## Findings

In Table 1 data are presented showing COVID-19 cases and deaths at 3 points in time – May 1, 2020; March 1, 2021; and September 1, 2021 by categories of county relative to the percent that voted for Trump in the 2020 presidential election. May 1, 2020 represents the early portion of the pandemic; March 1, 2021 is when vaccines were becoming available to large portions of the population, and September 1, 2021 is when the consequences of vaccination choices should be evident. Counties are divided into 5 categories relative to the percent of voters who cast their ballot for Donald Trump in the 2020 presidential election. These categories are: 1) counties where Trump received less than 25% of the vote; 2) counties where Trump received from 25 to less than 45% of the vote; 3) counties where Trump received from 45 to less than 55% of the vote; 4) counties where Trump received from 55 to less than 75% of the vote; and 5) counties where Trump received 75% or more of the vote.

**Table 1:**
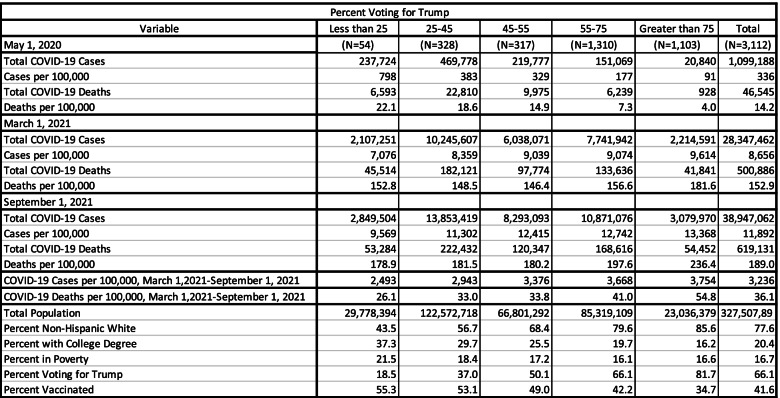
Covid-19 Cases and Deaths by Percent Fully Vaccinated by Percent Voting for Trump

Table 1 shows that, as expected, political views were strongly related to pandemic outcomes. Early in the pandemic, COVID-19 cases and deaths were much more extensive in counties where Trump received few votes. These counties tend to be large urban counties where the disease was concentrated in the first few months of the pandemic. This is evident because the number of residents per county is large. By March 1, 2021, this situation had reversed, and cases and deaths were more extensive in Trump leaning counties. After March 1, 2021 when vaccines were readily available, differences by political views became more pronounced. In the 6 months between March 1, 2021 and September 1, 2021, COVID-19 deaths increased by 26.1 per 100,000 residents in counties where Trump received less than 25% of the votes, while the rate of increase was more than twice as great (54.8 per 100,000 residents) in counties where Trump received more than 75% of the vote.

Table 1 also shows that vaccination rates were much lower in counties where Trump received a large portion of the vote. While 34.7% of residents were vaccinated in the average county where Trump received 75% or more of the vote, this proportion was much higher (55.3%) in the average county where Trump received less that 25% of the vote. Table 1 also shows that counties where Trump received a high share of votes tended to have a higher percent of non-Hispanic white residents, a lower proportion of adults with a college education and a lower share of residents in poverty.

Table 2 presents data showing that vaccination rates have significant implications for COVID-19 cases and deaths per 100,000 residents. For this table, counties have been broken into quartiles based on the percent of the population that is fully vaccinated. Early in the pandemic, the disease was centered in urban areas, where high proportions of the population would become later become vaccinated when it was available. By March 1, 2021, circumstances had completely flipped. Then between March 1, 2021 and September 1, 2021, COVID-19 cases and deaths increased much faster in counties with low vaccination rates than in counties with high vaccination rates, as expected. Table 2 also shows that counties with high vaccination rates had a lower percentage of Trump votes, a smaller proportion of non-Hispanic white residents, higher educational attainment levels, and lower poverty rates.

**Table 2:**
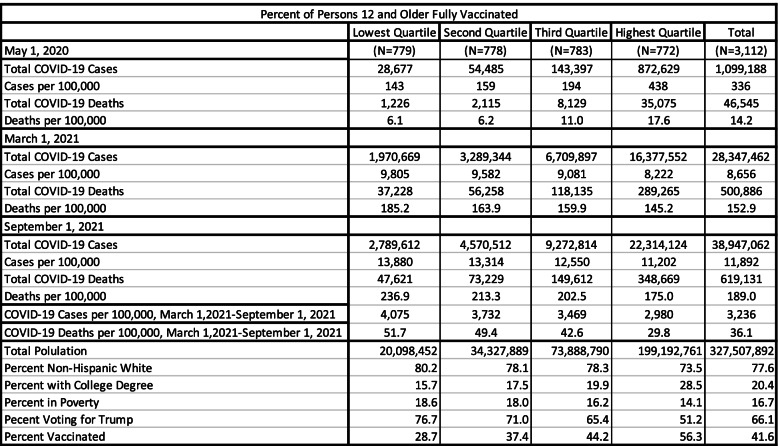
Covid-19 Cases and Deaths by Percent Fully Vaccinated

Path analysis results testing the research model developed for this study are presented in Table 3. The first panel of Table 3 shows the relationship between the exogenous variables and political views. As expected, all 3 variables are strongly related to political views and in the predicted direction. Counties most likely to vote for Trump included those with high proportions of non-Hispanic whites, low levels of educational attainment and low poverty rates. These 3 variables alone explain 70% of the variation in percent voting for Trump.

**Table 3:**
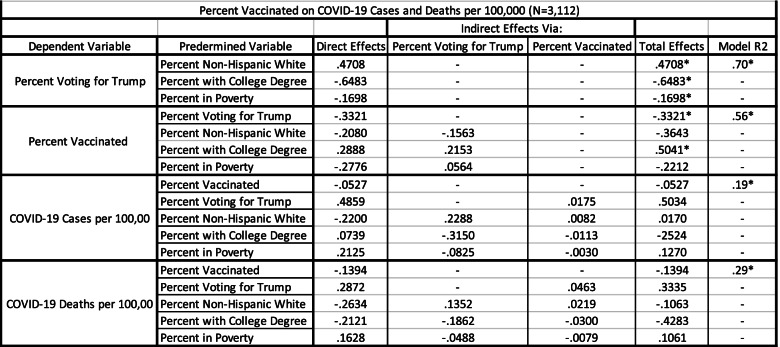
Total Effects, Indirect Effects, and Direct Effects of Independent Variables, Percent Voting for Trump and Percent Vaccinated on COVID-19 Cases and Deaths per 100,000 (*N* = 3112)

For the second panel, percent of persons fully vaccinated by county is the dependent variable. As expected, vaccination rates were inversely related to the percent of Trump voters (total effects = −.3321). The direct effect for the percent of non-Hispanic white residents on vaccine rates was negative. Additionally, there were further negative indirect effects through political views. Thus, the total effects were − .3643. Further, as predicted, the direct effect between educational attainment and vaccination levels was a positive .2888. Additionally, educational attainment positively impacts vaccination rates indirectly through political views as highly educated counties tended to have a lower percent of votes for Trump. The total effect was thus a very strong .5041. The direct effect between poverty rates and vaccination rates was negative, meaning persons in poverty are less likely to be vaccinated. Persons in poverty, however, are less likely to vote for Trump, which positively impacts vaccination rates. The total effects are still an inverse −.2212. Overall, these 4 variables explain 56% of the variation in vaccination rates.

**Fig. 2:**
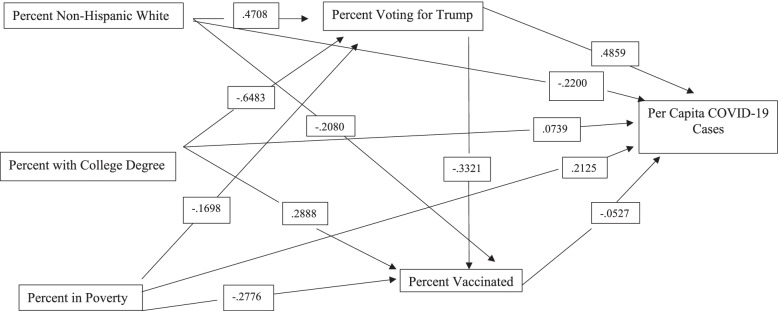
Relationships Between Variables Used in the Model and Per Capita COVID-19 Cases from March 1, 2021 to September 1, 2021

The next panel explores variations in COVID-19 cases per 100,000. These results are graphically presented in Fig. 2. Percent vaccinated is only weakly and inversely related to COVID-19 cases. The best predictor of COVID-19 cases was political views, where counties with a high proportion of Trump voters had much higher rates of COVID-19 cases. Counties with higher proportions of non-Hispanic white residents tended to have lower rates of COVID-19 cases. This negative direct effect was largely eliminated because of the positive indirect effects through political views, since counties with a high percent of non-Hispanic white residents were more likely to vote for Trump. Thus, the total effects were near zero. The total effects of educational attainment were strongly negative. This is mostly because of the indirect effects via political views since counties with high levels of educational attainment tend to cast a low percent of votes for Trump, which leads to fewer COVID-19 cases. Finally, counties with high poverty rates tended to have higher disease rates. The total effects were less than the direct effects because of the indirect effects via political views. High poverty counties tend not to vote for Trump in large numbers which indirectly results in fewer COVID-19 cases. Overall, the variables in the model explained 19% of the variation in COVID-19 cases per 100,000.

**Fig. 3:**
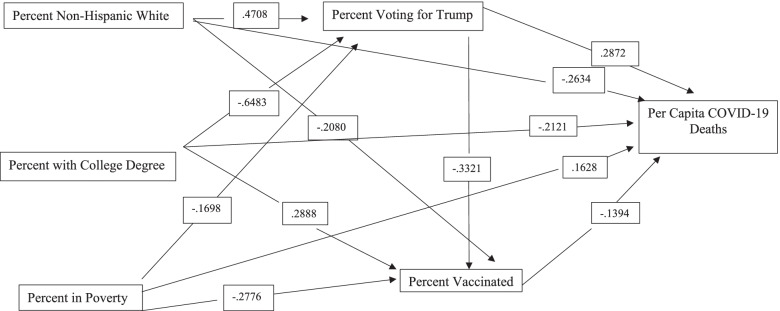
Relationships Between Variables Used in the Model and COVID-19 Deaths from March 1, 2021 to September 1, 2021

The final panel in Table 3 explores COVID-19 deaths per 100,000. Analysis results are graphically presented in Fig. 3. The strongest predictor of COVID-19 deaths was educational attainment. Counties with higher proportions of persons with a college degree had lower death rates. The direct effects for this relationship were substantial. Additionally, the indirect effects via political views were substantial as well since counties with high levels of educational attainment tend to cast a low percent of votes for Trump. Percent voting for Trump was also strongly and positively related to COVID-19 death rates. Death rates increase as the percent of votes for Trump increase. Direct effects show that counties with large non-Hispanic white populations had lower death rates. Because these counties tended to vote for Trump, which was positively related to COVID-19 deaths, the total effects were much weaker. Finally, poverty rates were positively related to COVID-19 death rates. In total, the variables in the model explained 29% of the variation in COVID-19 deaths.

## Conclusions

The data analyzed in this manuscript found strong support for the research model that had been developed. Three exogenous variables were strongly related to the percent voting for Trump. Percent voting for Trump was strongly and inversely related to percent vaccinated. As vaccination rates increased, COVID-19 cases and deaths per 100,000 tended to decline. Most significantly, not only were political views strongly related to vaccination rates, but also had important implications for COVID-19 cases and deaths. In Trump leaning counties, COVID-19 cases and deaths were more extensive than in counties where Trump received a lower percent of the vote. In counties where Trump received less than 25% of the vote, death rates per 100,000 were less than half as high as in counties where Trump received 75% or more of the vote between March 1, 2021 and September 1, 2021.

The consequences are profound. Because Republican political and thought leaders have downplayed the virus and failed to encourage vaccination, Republican leaning counties have failed to implement safety measures, failed to get a high proportion of residents vaccinated, and as a consequence suffered higher COVID-19 case and death rates. Combatting a virus should not be political. Political division has meant that the consequences of COVID-19 in the U.S. have been far worse than necessary. Most troubling, thousands of lives have been lost unnecessarily because people have not followed the advice of health experts. There is no question that the U.S. and the world will face future health or other crises. By reducing profound political divisions, we will be in much better position to address the problems, save lives and reduce economic and other costs. Misinformation is a problem everywhere, and this concern is amplified by social media [30]. A first and vital step in reducing divisions is restoring trust in science and health experts. In countries with multiple political parties, it may be possible for different parties to join forces to work for the common good, something that has been difficult to achieve in the U.S. where a two-party system is deeply entrenched. Regardless of how difficult it is to achieve it is critical that continued efforts be made to restore trust in science and health experts so that we can more effectively address the vital problems of today and those that will emerge in years to come.

## Data Availability

The data used in this article is available by contacting the author.

## References

[1] Brooks MM, Mueller JT, Thiede BC (2021). Rural-urban differences in the labor-force impacts of COVID-19 in the United States. Socius.

[2] Makridis C, Rothwell JT (2020). The real cost of political polarization: evidence from the COVID-19 pandemic.

[3] Pffefferbaum B, North CS (2020). Mental health and the COVID-19 pandemic. N Engl J Med.

[4] Yamin M (2020). Counting the cost of COVID-19. Int J Inf Technol.

[5] Hatchett RJ, Mecher CE, Lipsitch M (2007). Public health interventions and epidemic intensity during the 1918 influenza pandemic. Proc Natl Acad Sci.

[6] Lewis M (2021). The premonition.

[7] Morens DM, Fauci AS (2007). The 1918 influenza pandemic: insights for the 21^st^ century. J Infect Dis.

[8] Quammen D (2012). Spillover: animal infections and the next human pandemic.

[9] Quick JD, Fryer B (2018). The end of epidemics.

[10] Webster RG, Shortridge KF, Kawaoka Y (1997). Influenza: interspecies transmission and emergence of new pandemics. Immunol Med Microbiol.

[11] Loomis J (2018). Epidemics: the impact of germs and their power over humanity.

[12] Barry JM (2005). The great influenza.

[13] Diamond J (1999). Guns, germs and steel.

[14] Koch A, Brierley C, Maslin MM, Lewis SL (2019). Earth system impacts of the European arrival and great dying in the Americas after 1492. Quat Sci Rev.

[15] Yamey G, Schäferhoff M, Hatchett R, Pate M, Zhao F, McDade KK (2020). Ensuring global access to COVID-19 vaccines. Lancet.

[16] Graham BS (2020). Rapid COVID-19 Vaccine Development. Science.

[17] Isaacson W (2021). The code breaker: Jennifer Doudna, gene editing, and the future of the human race.

[18] Le TT, Andreadakis Z, Kumar A, Román RG, Tollefsen S, Saville M, Mayhew S (2020). The COVID-19 vaccine development landscape. Nat Rev Drug Discov.

[19] CDC (Center for Disease Control and Prevention). 2021. CDC.gov/coronavirus/2019*.*

[20] Thomas SJ, Moreira ED, Kitchin N, Absalon J, Gurtman A, Lockhart S (2021). Safety and efficacy of the BNT162b2 mRNA Covid-19 vaccine through 6 months. N Engl J Med..

[21] Fontanet A, Cauchemez S (2020). COVID-19 herd immunity: where are we?. Nat Rev Immunol.

[22] Asundi A, O’Leary C, Bhadelia N (2021). Global COVID-19 vaccine inequity: the scope, the impact, and the challenges. Cell Host Microbe.

[23] Callaghan T, Moghtaderi A, Lueck JA, Hotez PJ, Strych U, Dor A, Franklin Fowler E, Motta M (2020). Correlates and disparities of COVID-19 vaccine hesitancy.

[24] Malik AA, McFadden SM, Elharake J, Omer SB (2020). Determinants of COVID-19 vaccine acceptance in the US. EClinicalMedicine.

[25] Murphy J, Vallières F, Bentall RP, Shevlin M, McBride O, Hartman TK, McKay R, Bennett K, Mason L, Gibson-Miller J, Levita L (2021). Psychological characteristics associated with COVID-19 vaccine hesitancy and resistance in Ireland and the United Kingdom. Nat Commun.

[26] Peretti-Watel P, Seror V, Cortaredona S, Launay O, Raude J, Verger P, Fressard L, Beck F, Legleye S, l'Haridon O, Léger D (2020). A future vaccination campaign against COVID-19 at risk of vaccine hesitancy and politicization. Lancet Infect Dis.

[27] Sun Y, Monnat SM (2021). Rural-urban and within-rural differences in COVID-19 vaccination rates. J Rural Health..

[28] Dubé E, Vivion M, MacDonald NE (2015). Vaccine hesitancy, vaccine refusal and the anti-vaccine movement: influence, impact and “mplications”. Expert Rev Vacc.

[29] Jolley D, Douglas KM (2014). The effects of anti-vaccine conspiracy theories on vaccination intentions. Plos One.

[30] Bianco A, Mascaro V, Zucco R, Pavia M (2019). Parent perspectives on childhood vaccination: how to Deal with vaccine hesitancy and refusal?. Vaccine.

[31] Hornsey MJ, Finlayson M, Chatwood G, Begeny CT (2020). Donald Trump and vaccination: the effect of political identity, Conspiracist ideation and presidential tweets on vaccine hesitancy. J Exp Soc Psychol.

[32] Khubchandani J, Sharma S, Price JH, Wiblishauser MJ, Sharma M, Webb FJ (2021). COVID-19 vaccination hesitancy in the United States: a rapid national assessment. J Community Health.

[33] Offit PA (2005). The cutter incident, 50 years later. N Engl J Med.

[34] Andre FE, Booy R, Bock HL, Clemens J, Datta SK, John TJ, Lee BW, Lolekha S, Peltola H, Ruff TA, Santosham M (2008). Vaccination greatly reduces disease, disability, death and inequity worldwide. Bull World Health Organ.

[35] Jones AM, Omer SB, Bednarczyk RA, Halsey NA, Moulton LH, Salmon DA. Parents’ source of vaccine information and impact on vaccine attitudes, beliefs, and nonmedical exemptions. Adv Prev Med. 2012;2012:932741. 10.1155/2012/932741 PMC3469070 23082253

[36] Smith N, Graham T (2019). Mapping the anti-vaccination movement on Facebook. Inf Commun Soc.

[37] Hill HA, Elam-Evans LD, Yankey D, Singleton JA, Kang Y (2018). Vaccination coverage among children aged 19–35 months—United States, 2017. Morb Mortal Wkly Rep.

[38] Hotez P (2019). America and Europe’s new Normal: the return of vaccine-preventable diseases. Pediatr Res.

[39] Yang L, Grenfell BT, Mina MJ (2020). Waning immunity and re-emergence of measles and mumps in the vaccine era. Curr Opin Virol.

[40] Romer D, Jamieson KH (2020). Conspiracy theories as barriers to controlling the spread of COVID-19 in the US. Soc Sci Med.

[41] Albrecht DE (2019). The nonmetro vote and the election of Donald Trump. J Rural Social Sci.

[42] Goetz SJ, Davlasheridze M, Han Y, Fleming-Muñoz DA. Explaining the 2016 Vote for President Trump across U.S. Counties. Appl Econ Perspect Policy. 2018. 10.1093/aepp/ppy026.

[43] Monnat SM, Brown DL (2017). More than a rural revolt: landscapes of despair and the 2016 presidential election. J Rural Stud.

[44] Scala DJ, Johnson KM (2017). Political polarization along the rural-urban continuum? The geography of the presidential vote, 2000-2016. Ann Am Acad Polit Soc Sci.

[45] Jardina A. White identity politics: Cambridge: Cambridge University Press; 2019.

[46] Oberhauser AM, Krier D, Kusow AM (2019). Political moderation and polarization in the heartland: economics, rurality, and social identity in the 2016 U.S. presidential election. Sociol Q.

[47] Smith DN, Hanley E (2018). The anger games: who voted for Donald Trump in the 2016 election and why?. Crit Sociol.

[48] Tesler M, Sears DO (2010). Obama’s Race.

[49] Jardina A (2020). In-group love and out-group hate: white racial attitudes in contemporary US elections. Polit Behav..

[50] Dionne EJ (2016). Why the right went wrong.

[51] Aistrup J (1996). The southern strategy revisited: republican top-down advancement in the south.

[52] Aldrich JH (2000). Southern parties in state and nation. J Polit.

[53] Black E, Black M (2009). The rise of southern republicans.

[54] Farrell JA (2017). Richard Nixon.

[55] Phillips K (2015). The emerging republican majority: updated edition.

[56] Gilens M (1999). Why American hate welfare.

[57] Hochschild AR (2016). Strangers in their own land.

[58] Wetts R, Willer R (2018). Privilege on the precipice: perceived racial status threats Lead white Americans to oppose welfare programs. Soc Forces.

[59] McKee S (2008). Rural voters and the polarization of American presidential elections. Polit Sci Polit.

[60] Sides J, Tesler M, Vavreck L (2017). The 2016 US election: how trump lost and won. J Democr.

[61] Albrecht DE (2021). COVID-19 in rural America: impacts of politics and disadvantage. Rural Sociol..

[62] Dunlap RE, Van Liere KD, Mertig AG, Jones RE (2000). New trends in measuring environmental attitudes. J Soc Issues.

[63] Kahan DM, Peters E, Wittlin M, Slovic P, Ouellette LL, Braman D, Mandel G (2012). The polarizing impact of science literacy and numeracy on perceived climate change risks. Nat Clim Chang.

[64] McCright AM, Dunlap RE (2011). Cool dudes: the denial of climate change among conservative white males in the United States. Glob Environ Chang.

[65] Herring C (2009). Does diversity pay? Race, gender, and the business Case for diversity. Am Sociol Rev.

[66] Conway EM, Oreskes N. Why Conservatives Turned against Science. Chron Rev. 2012;November: 5.

[67] Gauchat G (2012). Politicization of science in the public sphere: a study of public Trust in the United States, 1974 to 2010. Am Sociol Rev.

[68] McCright AM, Dentzman K, Charters M, Diet T (2013). The influence of political ideology on trust in Science. Environ Res Lett.

[69] Mooney C (2007). The republican war on science.

[70] Oreskes N, Conway EM. Merchants of doubt: how a handful of scientists obscured the truth on issues from tobacco smoke to global warming: New York: Bloomsbury Publishing USA; 2011.

[71] Lichter DT, Parisi D, Taquino MC (2012). The geography of exclusion: race, segregation, and concentrated poverty. Soc Probl.

[72] Hughes MM, Wang A, Grossman MK, Pun E, Whiteman A, Deng L, Hallisey E, Sharpe JD, Ussery EN, Stokley S, Musial T (2021). County-level COVID-19 vaccination coverage and social vulnerability—United States, December 14, 2020–march 1, 2021. Morb Mortal Wkly Rep.

[73] Howell SE, Fagan D (1988). Race and trust in government: testing the political reality model. Pub Opin Q.

[74] Nunnally SC. Trust in black America: race, discrimination, and politics. New York: NYU Press; 2012.

[75] Moore JX, Gilbert KL, Lively KL, Laurent C, Chawla R, Li C, Johnson R, Petcu R, Mehra M, Spooner A, Kolhe R (2021). Correlates of COVID-19 vaccine hesitancy among a community sample of African Americans living in the southern United States. Vaccines.

[76] Adhikari S, Pantaleo NP, Feldman JM, Ogedegbe O, Thorpe L, Troxel AB (2020). Assessment of community-level disparities in coronavirus disease 2019 (COVID-19) infections and deaths in large US metropolitan areas. JAMA Netw Open.

[77] Cheng KJG, Sun Y, Monnat SM (2020). COVID-19 death rates are higher in rural counties with larger shares of blacks and Hispanics. J Rural Health.

[78] Jung J, Manley J, Shrestha V (2020). Coronavirus infections and deaths by poverty status: the effects of social distancing. J Econ Behav Organ..

[79] Case A, Deaton A (2020). Deaths of despair.

[80] Chokshi DA (2018). Income, poverty, and health inequality. JAMA.

[81] Bruine de Bruin W, Saw HW, Goldman DP. Why Conservatives Turned against ScienceWhy Conservatives Turned against Science. J Risk Uncertainty. 2020. 10.1007/s11166-020-09336-3.

[82] Hamilton LC, Safford T. Conservative media consumers less likely to Wear masks and less worried about COVID-19: Carsey Perspectives. Durham: University of New Hampshire; 2020.

[83] Barrios JM, Hochberg Y. Risk Perception Through the Lens of Politics in the Time of COVID-19 Pandemic. Cambridge: National Bureau of Economic Research Working Paper 27008; 2020.

[84] Calvillo DP, Ross BJ, Garcia RJ, Smelter TJ, Rutchick AM (2020). Political ideology predicts perceptions of the threat of COVID-19 (and susceptibility to fake news about it). Soc Psychol Personal Sci.

[85] Hill T, Gonzalez KE, Davis A (2020). The nastiest question: does population mobility vary by state political ideology during the novel coronavirus (COVID-19) pandemic?. Sociol Perspect..

[86] Hill T, Gonzalez KE, Burdette A (2020). The blood of Christ compels them: state religiosity and state population mobility during the coronavirus (COVID-19) pandemic. J Relig Health.

[87] Perry SL, Whitehead AL, Grubbs JB (2020). Culture wars and COVID-19 conduct: Christian nationalism, religiosity, and Americans’ behavior during the coronavirus pandemic. J Sci Study Relig.

[88] Lin C, Tu P, Beitsch LM (2021). Confidence and receptivity for COVID-19 vaccines: a rapid systematic review. Vaccines.

[89] Viswanath K, Bekalu M, Dhawan D, Pinnamaneni R, Lang J, McLoud R (2021). Individual and social determinants of COVID-19 vaccine uptake. BMC Public Health.

[90] Salt Lake Tribune (2021). Utah Protest Against ‘Medical Tyranny’ Includes Burning a Giant Effigy of a Vaccine Syringe.

[91] Rutjens BT, van der Linden S, van der Lee R (2021). Science skepticism in times of COVID-19. Group Process Intergroup Relat.

[92] Paz C. All the President’s lies about the coronavirus. Boston: The Atlantic; 2020.

[93] Allcott H, Boxell L, Conway J, Gentzkow M, Thaler M, Yang D. Polarization and Public Health: Partisan Differences in Social Distancing During the Coronavirus Pandemic. Cambridge: National Bureau of Economic Research Working Paper 26946; 2020. 10.1016/j.jpubeco.2020.104254 PMC7409721 32836504

[94] Fridman A, Gershon R, Gneezy A (2021). COVID-19 and vaccine hesitancy: a longitudinal study. Plos One.

[95] Desmet K, Wacziarg R. Understanding spatial variation in COVID-19 across the United States. J Urban Econ. 2021;1:103332. 10.1016/j.jue.2021.103332 PMC7948676 33723466

[96] New York Times (2021). NYTimes/COVID-19-data.

[97] New York Times (2020). NYTimes/election-data.

